# Spatiotemporal Patterns and Driving Factors of Water Self-Purification Capacity in the Source Area of the Western Route of the South-to-North Water Diversion

**DOI:** 10.3390/toxics14100896

**Published:** 2026-10-10

**Authors:** Fei Xing, Hongbin Lu, Bingfei Yan, Shuhu Xiao

**Affiliations:** 1 State Key Laboratory of Environmental Criteria and Risk Assessment, Chinese Research Academy of Environmental Sciences, Beijing 100012, China; feixgy@126.com (F.X.); ybf201506@163.com (B.Y.); 2 State Environmental Protection Key Laboratory of Estuarine and Coastal Environment, Chinese Research Academy of Environmental Sciences, Beijing 100012, China; 3 State Key Laboratory of Coal Conversion, Institute of Coal Chemistry, Chinese Academy of Sciences, Taiyuan 030001, China; luhongbin@sxicc.ac.cn

**Keywords:** Western Route of the South-to-North Water Diversion Project, the water self-purification capacity, water source areas, the nitrogen removal, the phosphorus removal

## Abstract

The water self-purification capacity in the source area of the Western Route of the South-to-North Water Diversion Project of China was investigated. This research aimed to promote the coordinated development of water source protection and water diversion benefits. Based on water quality tests conducted in the upstream section of the water source area, the results indicate that the water quality in the upper reaches of the water source region is generally good. However, total nitrogen at some sites in the Dadu River basin exceeded 1.0 mg/L, and the mean total nitrogen concentration in the Dadu River basin was higher than that in the Yalong River basin, while total phosphorus levels remained low in both basins. The nitrogen removal of the Yalong River Basin and the Dadu River Basin attained a peak of 34,654 t in 2012. The nitrogen removal was more than ten times that of phosphorus. Among the prefectures, the water self-purification service value ranks as follows: Garzê > Liangshan > Aba > Golog. The correlations between water quality indicators and driving factors-including land use types, GDP, precipitation, population, and temperature were discussed. In terms of spatial drivers, barren land (r = 0.41) and grassland (r = 0.47) are the major land-use contributors to TN. The model was found which can be used to quantify management priorities and predict potential risks associated with nitrogen pollution. After water diversion, the removal of nitrogen and phosphorus in the water source area declined, which was not linearly related to the amount of water diverted.

## 1. Introduction

Sufficient water resources of good quality constitute the physical basis for achieving high-quality economic growth [1]. This significant water-diversion project, known as the South-to-North Water Diversion Project (SNWDP), aims to remedy the geographical disparity in water endowments that has long plagued northern China [2,3,4]. Consisting of the East, Middle, and West routes [5], the SNWDP channels water from the water-abundant southern regions to the dry north [6] and has been instrumental in augmenting groundwater storage [7] as well as alleviating flood hazards in the wet season [8].

Human activities are not only intensifying land-use change [9] but also exerting profound impacts on biogeochemical cycles [10] and hydrological processes [11]. Meanwhile, water diversion projects modify flow regimes and promote nutrient enrichment [1]. Collectively, these pressures drive water quality variability, potentially triggering eutrophication [10], degrading aquatic biodiversity [11], leading to the accumulation of toxic substances such as emerging contaminants, and ultimately heightening water security risks [12]. In response, enhanced long-term monitoring of source-water quality is urgently required, alongside robust projections of future land-use dynamics and self-purification capacity under continued anthropogenic forcing. Such efforts are essential for safeguarding water security and guiding the sound implementation of water diversion schemes.

The Western Route Project is designed to transfer water from the Yalong and Dadu rivers to the Tao River, so as to relieve water stress in the arid northwest. The source area, situated in the upper reaches, experiences relatively low anthropogenic impact, and available measurements confirm that upstream water quality surpasses that of downstream reaches [13]. To date, most investigations into the water quality of the Eastern and Middle Routes of the South-to-North Water Diversion have been initiated after project commissioning [11,14], with evidence pointing to eutrophication events following operation [11]. Routine monitoring relies on discrete sampling along the routes; however, such a point-based approach fails to deliver a holistic assessment, thereby limiting our ability to decipher the drivers of water quality change. Given these challenges, robustly assessing the basin’s self-purification capacity is essential for optimizing regional water allocation strategies.

The water self-purification service refers to the capacity of vegetation and soil to reduce and remove nutrients such as nitrogen and phosphorus through transfer, transport, and storage, thereby purifying water bodies from non-point source pollution [15]. Methods for evaluating ecosystem service value of water self-purification capacity include the emergy method [16,17], the value quantification method [18,19], and the InVEST model [20,21], among others. Although the value-equivalent method is valued for its low data requirements and simplicity, it is a broad-scale approach intended for regional applications, and its accuracy tends to decrease when applied to smaller spatial units [22]. Since the parameters of the emergy method are generated through the computation of ecosystem matter and energy fluxes, they are fairly intricate [23]. Widely applied in ecosystem service assessments, the InVEST model proves robust for provisioning, regulating and supporting services and is also capable of multi-scenario simulations [24,25]. The InVEST model demands relatively complete input data and ensures greater accuracy in its computations.

Ecosystem patterns and the provision of services are largely driven by land use change, the dynamic processes of which directly alter ecosystem structure and function [15,26]. Thus, the prediction of future trends is critical for the design of sustainable strategies. In this context, land use scenario simulation serves as a key tool for informing regional ecological decisions [27]. Prevalent models for land use change forecasting comprise the Cellular Automaton-Markov (CA-Markov) [28], the Future Land Use Simulation [29], and the Patch-Generating Land Use Simulation (PLUS) approaches [30].

The PLUS model provides more refined simulations and can identify non-linear land use dynamics. Therefore, this study employs the PLUS and InVEST models and the replacement cost method to assess the water self-purification capacity of the water source area. The study conducted field sampling and water quality testing in the upper reaches of the Yalong River and Dadu River basins. The temporal evolution of the water self-purification capacity of the water source area was analyzed, the main driving factors of water quality changes were identified, and regression equations were established. Furthermore, based on the PLUS model, we conducted scenario simulations of water quality conditions in 2050 to predict the future trends of water self-purification changes under anthropogenic disturbances. By calculating the ecological service value of water self-purification in the western route water source area, we converted intangible ecological functions—such as water conservation and pollutant reduction—into quantifiable economic indicators. This provides an objective basis for formulating ecological compensation standards, offers scientific support for optimizing water transfer scale and operation schedules, quantifies the ecological gains/losses, balances the trade-offs among different services, and lays the data foundation for project impact assessments and green hydropower certification. Ultimately, this research aimed to promote the coordinated development of water source protection and water diversion benefits.

## 2. Materials and Methods

### 2.1. Study Area

**Figure 1:**
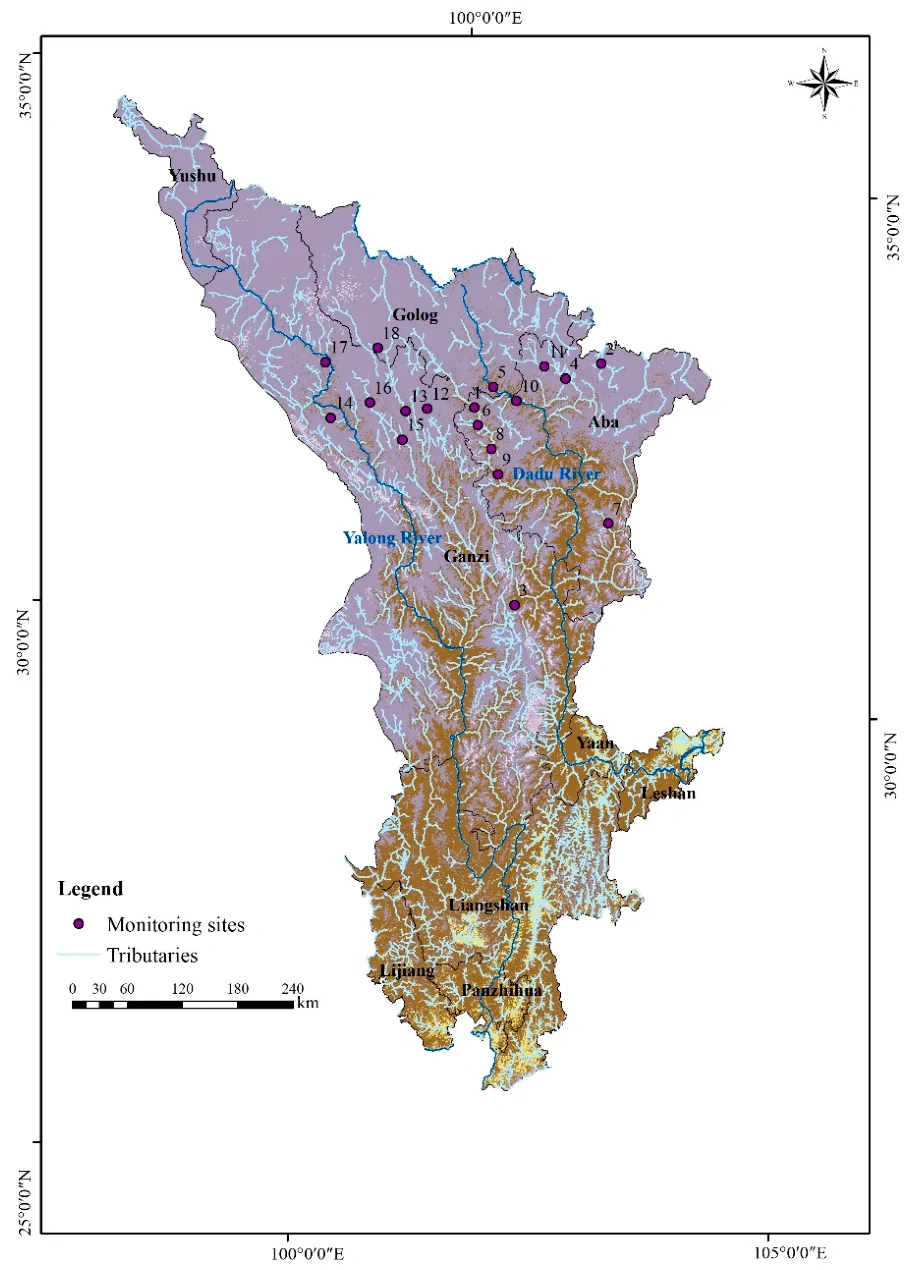
The study area and water quality monitoring points.

The West Route Project’s water source lies on the eastern flank of the Qinghai-Tibet Plateau, as shown in Figure 1, where ecosystems show clear vertical belts. The plateau is hilly, averaging 4200–4700 m in altitude. The ecological environment is cold and fragile, where the vegetation is predominantly grassland and meadow. The cities and prefectures in the study area are consistent with those in the article [31].

### 2.2. Water Quality Analysis

The environmental quality standards for surface water (GB3838-2002), a national standard of China, stipulate the permissible limits and analytical procedures for a range of water quality indicators—including pH, electrical conductivity (EC), total dissolved solids (TDS), ammonia nitrogen (NH_3_-N), chemical oxygen demand by potassium permanganate (COD_Mn_), total phosphorus (TP), total nitrogen (TN), and conductivity (Cond)—applicable to various categories of surface water [32]. A total of 18 monitoring sites were established in the upstream sections of the Yalong and Dadu rivers in 2022 within the source water area, where water quality parameters including pH, EC, TDS, COD, NH_3_-N, TN, and TP were determined.

Water samples were collected in polyethylene bottles. The pH and electrical conductivity were measured using a portable meter (Mettler Toledo, Greifensee, Switzerland), and the TDS was measured using a portable meter (Milwaukee Instruments, Rocky Mount, NC, USA). The water samples were acidified with H_2_SO_4_ to pH ≤ 2 for the determination of other indicators. The COD was determined by the dichromate reflux method. Ammonia nitrogen was analyzed using a spectrophotometer with Nessler’s reagent method. The total nitrogen was analyzed by the alkaline potassium persulfate digestion-UV spectrophotometric method, and the total phosphorus was measured by persulfate digestion followed by colorimetric analysis using the molybdenum blue method.

### 2.3. Water Self-Purification Ecosystem Service Value

In this study, the nutrient delivery ratio (NDR) module of the InVEST model was applied to calculate the purification capacity of the watershed ecosystem for nitrogen and phosphorus [15]. The input data required for the NDR module include land use type, digital elevation model (DEM), nutrient runoff proxy data, nitrogen and phosphorus loading coefficients, vegetation nutrient retention ratio and critical distance, flow accumulation threshold, and the Borselli parameter [33,34].

(1) Land use data for the watershed were obtained from the CLCD (China Land Cover Dataset) database of Wuhan University [18], which provides annual land use datasets for China from 2002 to 2022 at a spatial resolution of 30 m × 30 m. Using the mask extraction tool in ArcGIS 10.4, we extracted the land use raster datasets for the watershed for the years 2002, 2007, 2012, 2017, and 2022, and then resampled them to obtain watershed land use datasets with a spatial resolution of 30 × 30 m.

(2) The DEM data [16] were derived from the SRTM3 data available from the Geospatial Data Cloud (geodata.cn), with a spatial resolution of 30 m × 30 m. After resampling and mask extraction using ArcGIS 10.4, we obtained the watershed DEM data at a resolution of 30 × 30 m.

(3) The nutrient runoff proxy data represent the level of nutrient transport in the watershed, and precipitation was used as a substitute in this study. The precipitation data [16] for the watershed were obtained from the National Earth System Science Data Center (geodata.cn), including raster datasets of precipitation for the years 2002, 2007, 2012, 2017, and 2022. After resampling and mask extraction using ArcGIS 10.4, we obtained the watershed precipitation datasets at a spatial resolution of 30 × 30 m.

The raw data were sequentially input into the nutrient delivery ratio module of the InVEST model to calculate the water self-purification capacity of nitrogen and phosphorus removal for the years 2002, 2007, 2012, 2017, and 2022.

The economic value of the watershed water self-purification service was calculated using the alternative engineering cost method, and the calculation formula was as follows:

(1) $${RV}_{wp}=Q_{N}\times P_{N}+Q_{P}\times P_{P}$$

where: *RV*_WP_ was the value of water self-purification service; *Q*_N_ was the nitrogen removal of the watershed ecosystem simulated by the InVEST model (kg); *P*_N_ was the treatment cost of nitrogen (yuan/kg); *Q*_P_ was the phosphorus removal of the watershed ecosystem simulated by the InVEST model (kg); *P*_P_ was the treatment cost of phosphorus (yuan/kg). According to the economic characteristics of the watershed, *P*_N_ and *P*_P_ were 1.5 yuan/kg and 2.5 yuan/kg, respectively.

### 2.4. Future Land Use Simulation

The land use expansion data for the period 2000–2020 were first acquired. The Land Expansion Analysis Strategy module was then employed to analyze the expansion patterns, using DEM, slope, GDP, and highway distribution as driving factors [35]. Subsequently, the CA-based Markov chain with random forest module [36] was used to project land use for 2050, based on the calculated probabilities of land development. To explore the future impacts, four land use scenarios were designed: natural change, dead water level, 4 billion m^3^ water transfer, and 8 billion m^3^ water transfer. Finally, the variation in the value of water self-purification ecosystem services in the source area under these future diversion scenarios was evaluated and discussed.

### 2.5. Statistical Methods

Statistical analyses were performed using SPSS (version 23). Pearson correlation analysis was used to examine the relationships among variables, and linear regression analysis was applied to explore the quantitative relationships between variables. The significance level was set at * *p* ≤ 0.05, ** *p* ≤ 0.01, *** *p* ≤ 0.001. The data followed a normal distribution.

## 3. Results and Discussion

### 3.1. Water Quality in the Water Source Area

**Figure 2:**
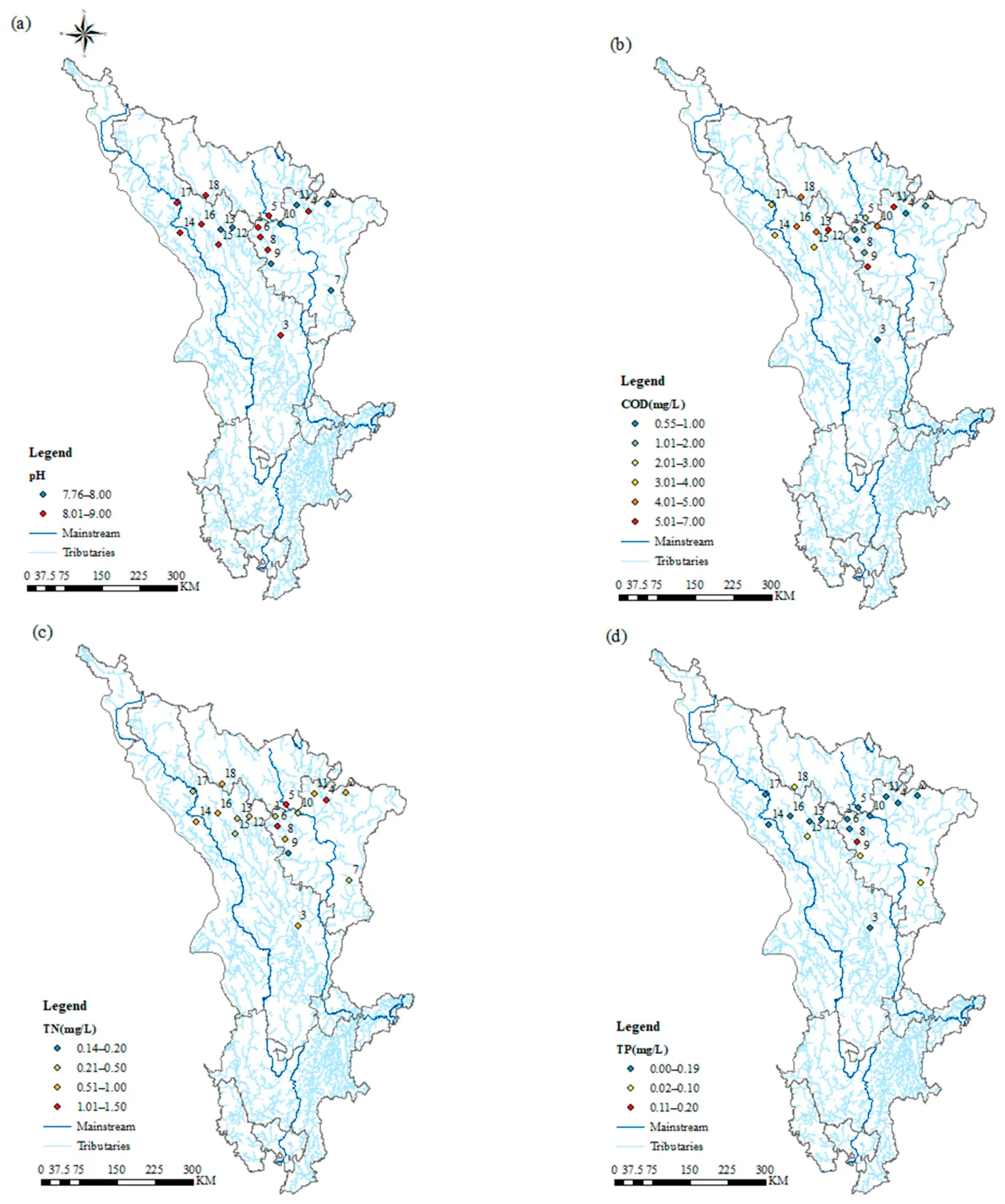

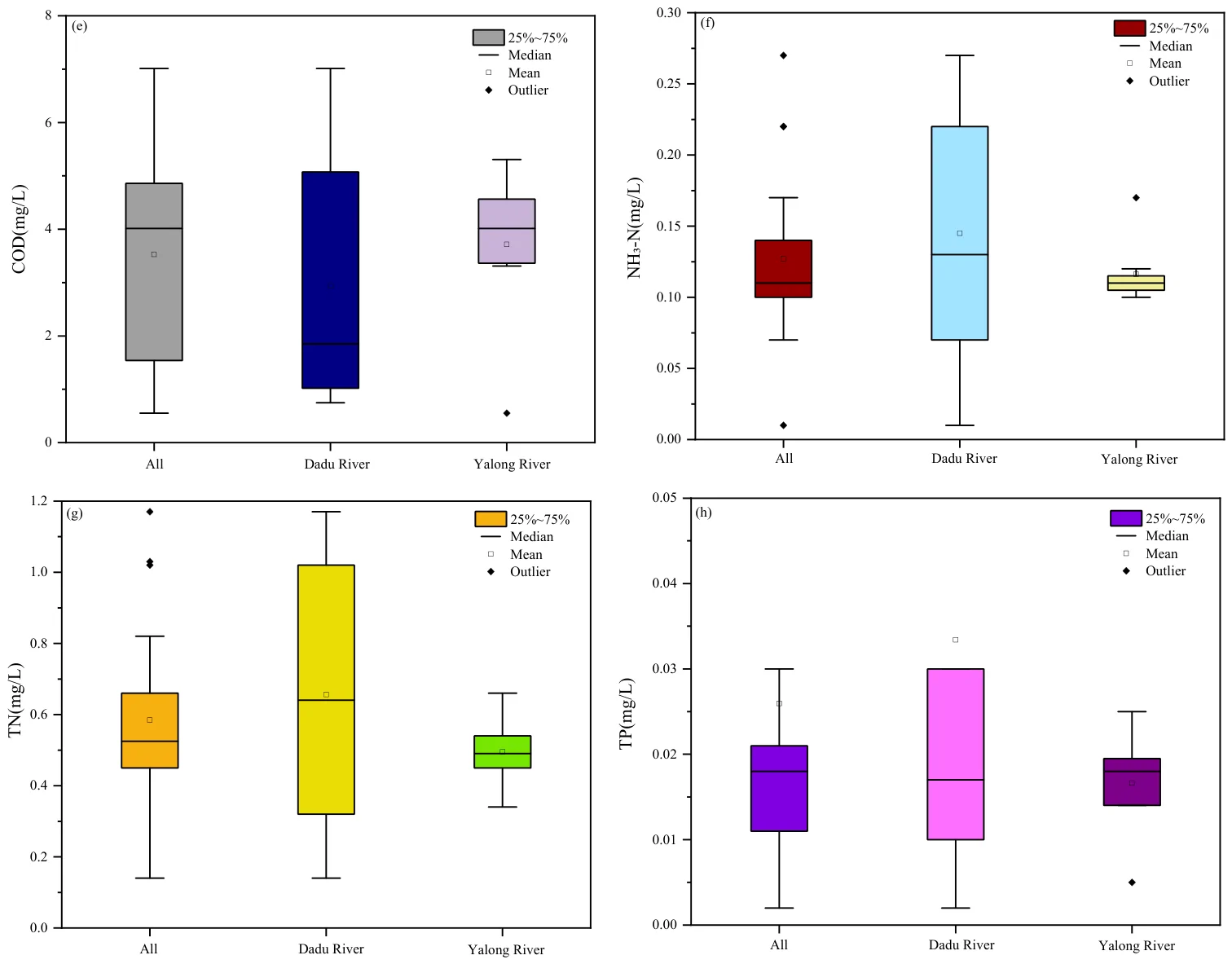
The water quality conditions in the upper reaches of the Yalong River and the Dadu River: (**a**) pH value; (**b**) COD value; (**c**) TN value; (**d**) TP value; (**e**) the box plot of COD; (**f**) the box plot of NH_3_-N; (**g**) the box plot of TN; (**h**) the box plot of TP.

The upstream area of the water source region is designated for reservoir construction. Water samples were collected from key sites along the rivers within the water source region during the wet season in June, July, and September 2022. The average values of the water quality test results were shown in Table S1. The water quality conditions in the upper reaches of the Yalong River and the Dadu River are shown in Figure 2a–h. The average pH value of the water body in the upper reaches of the water source region was 8.12. The COD values ranged from 0.52 mg/L to 7.01 mg/L, with an average of 3.28 mg/L, indicating relatively low COD levels in the upstream water. The average ammonia nitrogen concentration was low at 0.13 mg/L. Total nitrogen values ranged from 0.14 mg/L to 1.17 mg/L, with an average of 0.58 mg/L; at some sampling sites along the Dadu River, the values exceeded 1.0 mg/L. The average total phosphorus concentration was low at 0.03 mg/L.

In the Yalong River basin, the average pH value of the water body was 8.10. COD values ranged from 0.55 mg/L to 5.30 mg/L, with an average of 3.72 mg/L. The average ammonia nitrogen concentration was low at 0.12 mg/L. Total nitrogen values ranged from 0.34 mg/L to 0.66 mg/L, with an average of 0.50 mg/L. The average total phosphorus concentration was low at 0.02 mg/L.

In the Dadu River basin, the average pH value of the water body was 8.13. COD values ranged from 0.75 mg/L to 7.01 mg/L, with an average of 2.94 mg/L. The average ammonia nitrogen concentration was low at 0.15 mg/L. Total nitrogen values ranged from 0.14 mg/L to 1.17 mg/L, with an average of 0.66 mg/L. The average total phosphorus concentration was low at 0.03 mg/L.

Overall, the water quality in the upper reaches of the water source region is generally good. However, total nitrogen at some sites in the Dadu River basin exceeded 1.0 mg/L, and the mean total nitrogen concentration in the Dadu River basin was higher than that in the Yalong River basin, while total phosphorus levels remained low in both basins. The total dissolved solids (TDS) content in the upstream area of the water source was 73 mg/L–206 mg/L, indicating a low risk of soil salinization [1].

Sampling sites were established in the middle and upper reaches of the Tao River, Laqu River, Yalong River Bridge, Niqu River, Duqu River at Zhu’anda, and Make River. The results showed that the concentrations of COD, sulfide, dissolved oxygen, and ammonia nitrogen generally meet Class I or Class II surface water quality standards. Total nitrogen in the middle and upper reaches of the Tao River and Jiaqu River meets Class III standards, while in areas such as the Duqu River at Zhu’anda and the Make River, it even reached Class IV standards. Total phosphorus in localized sections of the Make River meets Class III standards. The levels of nitrogen and phosphorus in the water are the primary drivers of downstream eutrophication, directly controlling and affecting the health of the entire downstream aquatic ecosystem. Therefore, paying close attention to the water quality, especially the nitrogen and phosphorus content, in the source area is crucial for maintaining the balance of the aquatic ecosystem and for rationally setting reservoir discharge flows during the normal and dry seasons.

### 3.2. Variation Pattern of Water Purification in the Water Source Area

**Figure 3:**
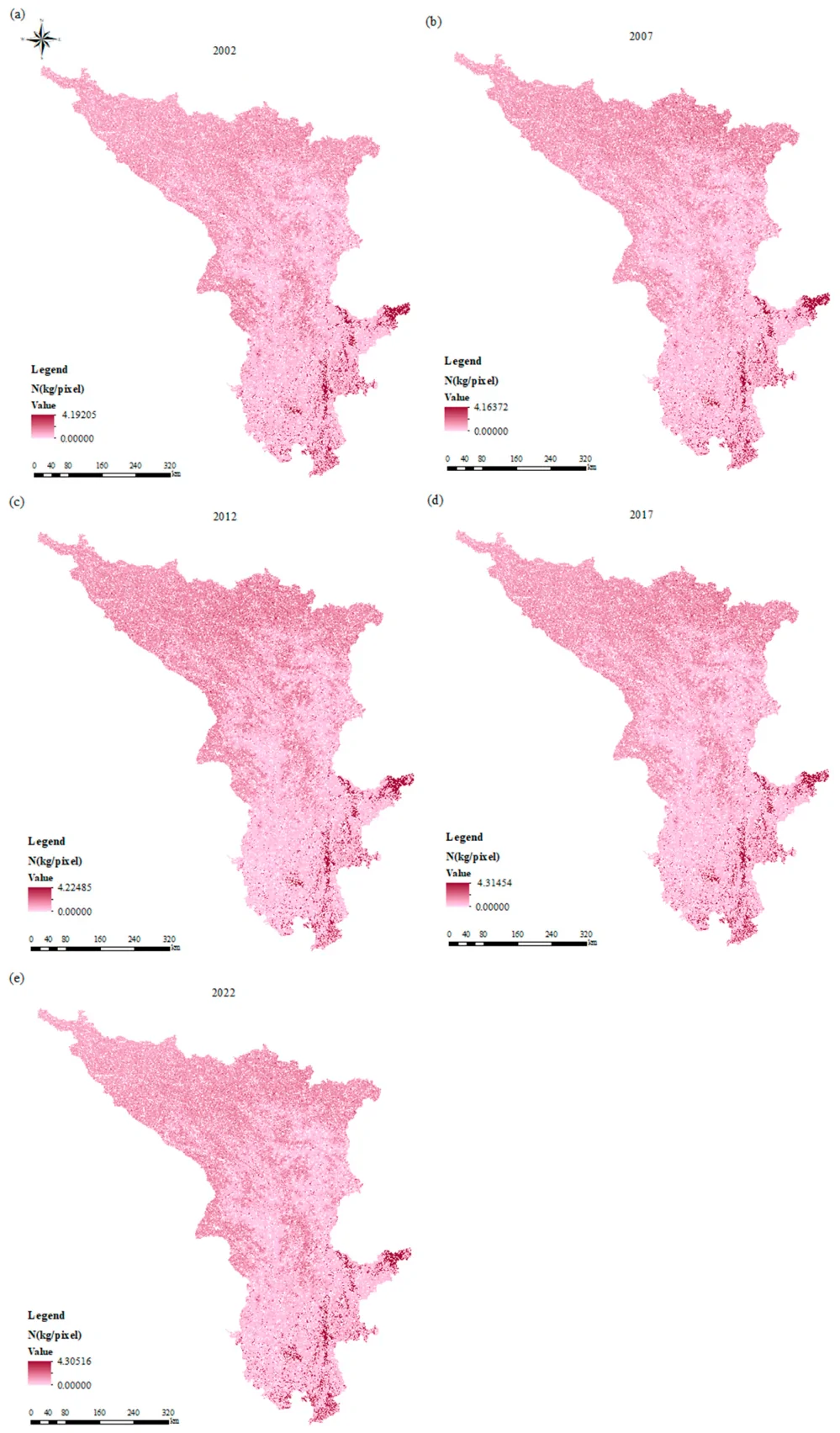
The spatial distribution of nitrogen removal in the water source area in 2002, 2007, 2012, 2017 and 2022: (**a**) 2002; (**b**) 2007; (**c**) 2012; (**d**) 2017; (**e**) 2022.

The water self-purification capacities of the Yalong River Basin and the Dadu River Basin from 2002 to 2022 were investigated. The spatial distribution of nitrogen removal in the water source area in 2002, 2007, 2012, 2017 and 2022 was shown in Figure 3a–e. The nitrogen removal from 2002 to 2022 was 33,300 t, 33,660 t, 34,654 t, 33,942 t, and 33,400 t, respectively. Overall, nitrogen removal in the water source area exhibited a trend of first increasing and then decreasing, reaching a maximum of 34,654 t in 2012. Over the 20-year period, the nitrogen purification value of the basins ranged from 49.9 million to 52.0 million yuan.

**Figure 4:**
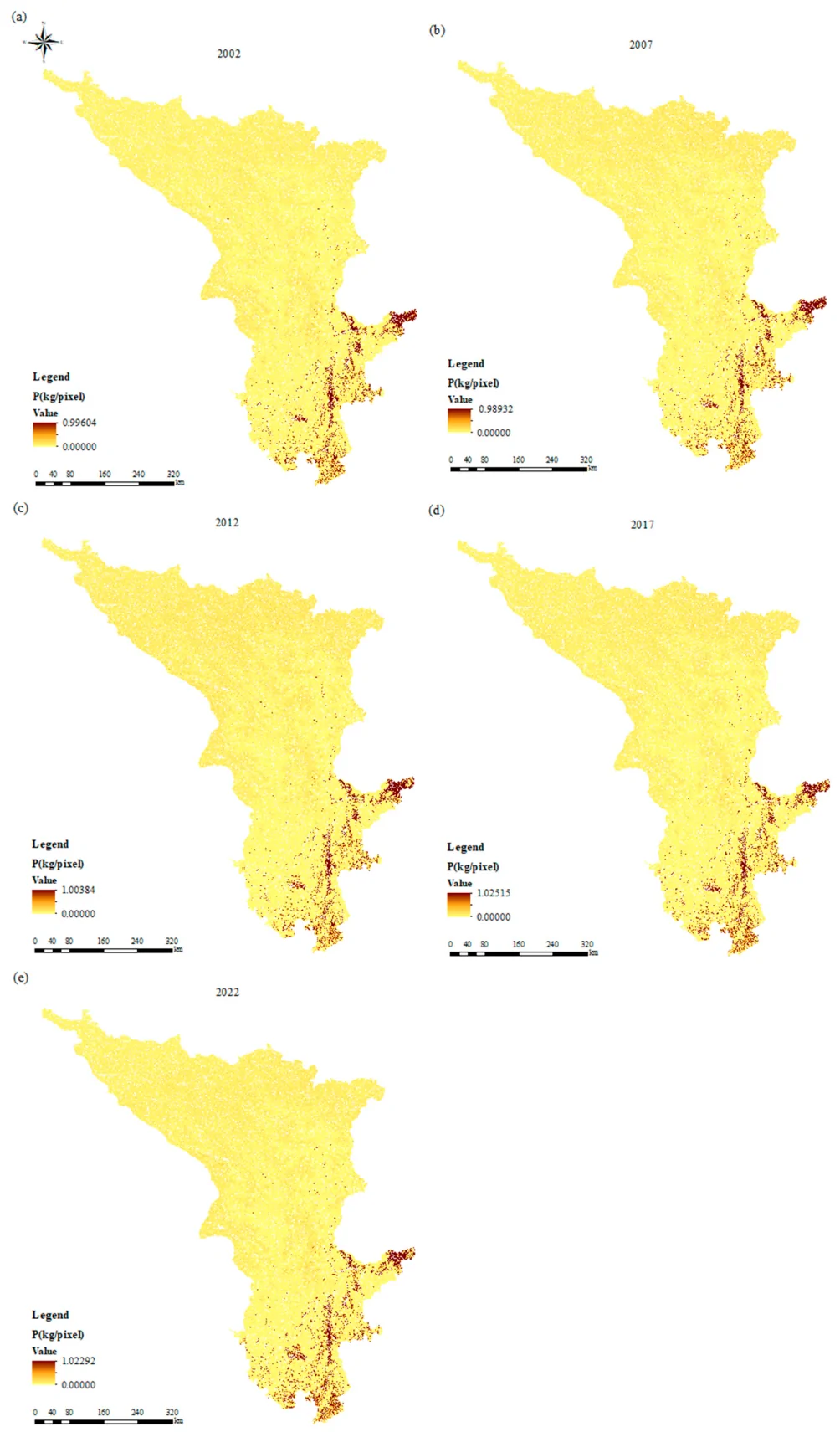
The spatial distribution of phosphorus removal in the water source area of 2002, 2007, 2012, 2017 and 2022 (**a**) 2002; (**b**) 2007; (**c**) 2012; (**d**) 2017; (**e**) 2022.

The spatial distribution of phosphorus removal in the water source area in 2002, 2007, 2012, 2017, and 2022 was shown in Figure 4a–e. The phosphorus removal from 2002 to 2022 was 3283 t, 3249 t, 3250 t, 3306 t, and 3281 t, respectively. Phosphorus removal showed fluctuating changes, peaking at 3306 t in 2017. Nitrogen removal was more than ten times that amount of phosphorus, indicating that basin management efforts have primarily targeted nitrogen. The phosphorus purification value ranged from 8.1 million to 8.3 million yuan in 20 years.

The overall water self-purification capacity of the basins varied between 58.2 million and 60.1 million yuan. The purification value of nitrogen was about six times than that of phosphorus, showing that the water self-purification capacity of the water source area was mainly dominated by nitrogen removal. The spatial distribution pattern of water self-purification capacity in the watershed has shown little change over time, which is related to the long-term land cover pattern changes within the watershed.

**Figure 5:**
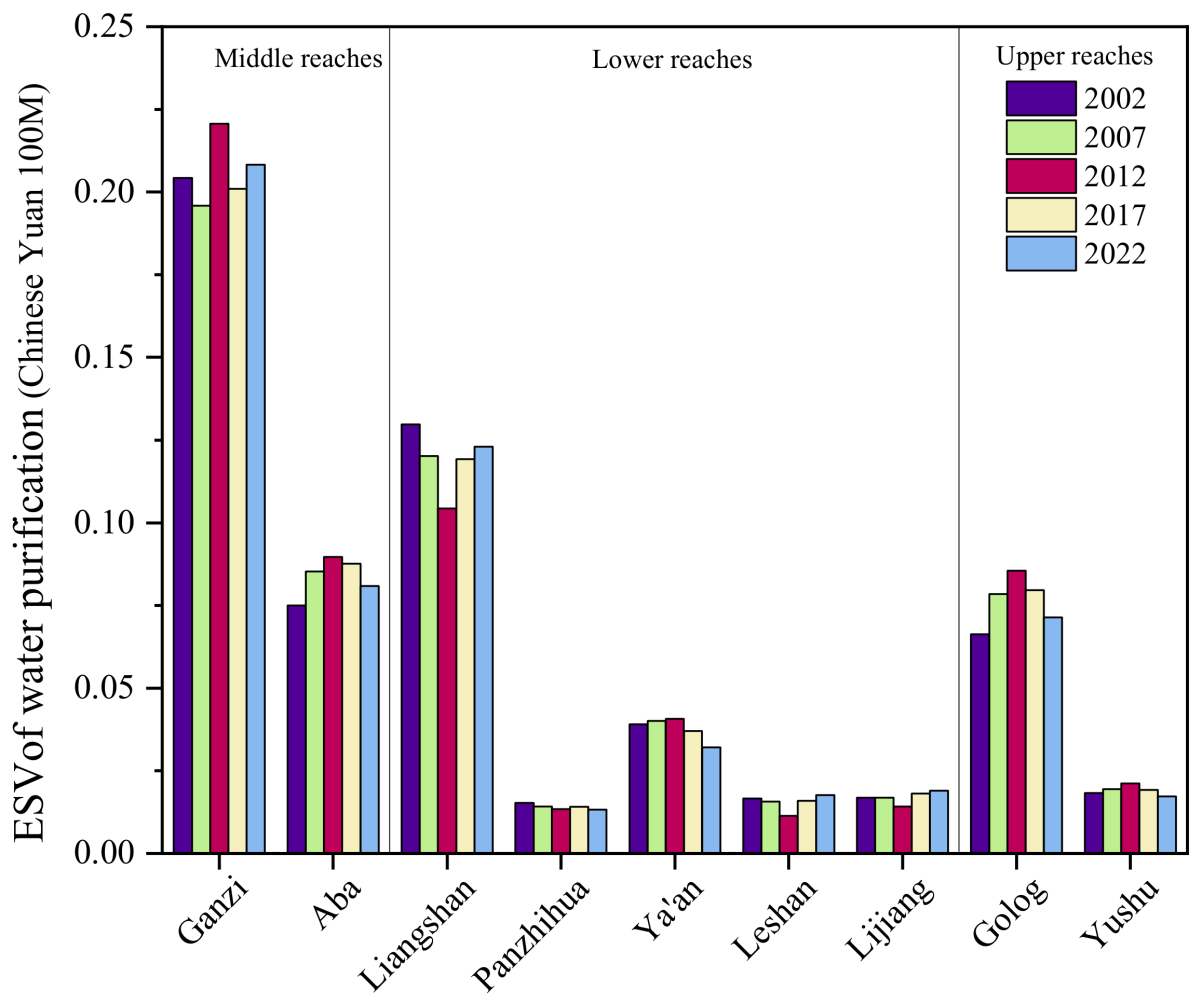
Water self-purification ecosystem service value in the water source area.

The distribution of water self-purification service values across the upper, middle, and lower reaches of the river basins is shown in Figure 5. The upper reaches mainly included Yushu Prefecture and Golog Prefecture; the middle reaches mainly included Garzê Prefecture and Aba Prefecture; and the lower reaches mainly included Liangshan Prefecture, Panzhihua City, Ya’an City, Leshan City, and Lijiang City. The order of water self-purification values across the basins was: middle reaches > lower reaches > upper reaches. The water quality in the upper reaches was relatively good, while the middle and lower reaches have greater capacity for nitrogen removal. Among the prefectures, the water self-purification service value ranks as follows: Garzê > Liangshan > Aba > Golog.

Over the 20-year period, the water self-purification service value ranged from 84.5 million to 106.7 million yuan in the upper reaches, from 279.3 million to 310.2 million yuan in the middle reaches, and from 184.2 million to 217.7 million yuan in the lower reaches. Within the upper reaches, Golog had a relatively high value of 66.3 million to 85.5 million yuan. In the middle reaches, both Garzê and Aba had high values, with Garzê reaching 195.8 million to 208.3 million yuan and Aba ranging from 75.0 million to 89.6 million yuan. In the lower reaches, Liangshan had the highest value, at 104.3 million to 129.8 million yuan. Garzê Prefecture and Aba Prefecture played an important role in nitrogen and phosphorus removal.

### 3.3. Analysis of Driving Factors

**Figure 6:**
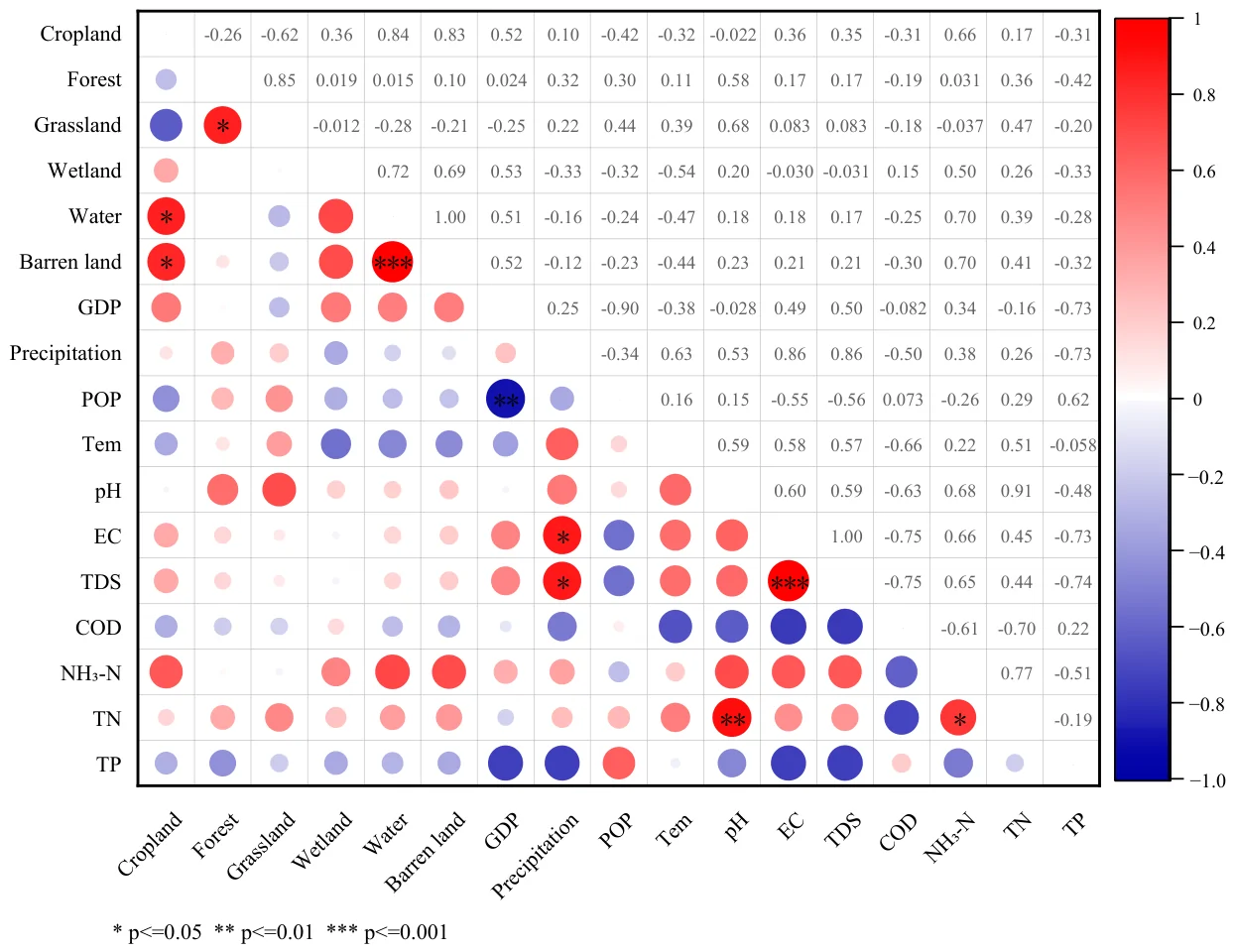
The correlations between water quality indicators and driving factors.

The correlations between water quality indicators and driving factors-including land use types, GDP, precipitation, population, and temperature are presented in Figure 6. For NH_3_-N, relatively high correlations are observed with cropland (r = 0.66), water bodies and barren land (both r = 0.70), and GDP (r = 0.34). Based on the ratio of ammonium nitrogen to total nitrogen (0.11–0.68), one of the nitrogen forms indicating recent nitrogen pollution of the water body is ammonium nitrogen.

For total nitrogen, one of its dominant forms may be ammonia nitrogen (r = 0.77). The increase in TN significantly drives the rise in water pH (r = 0.91), indicating that the study area faces a considerable risk of eutrophication. In terms of spatial drivers, barren land (r = 0.41) and grassland (r = 0.47) are the major land-use contributors to TN, which may be associated with soil erosion from exposed surfaces and fertilized grassland areas. Notably, COD exhibits a significant negative correlation with TN (r = −0.70), suggesting either a strong denitrification process in the water body or the influence of external tailwater recharge that has undergone nitrogen removal treatment. Compared to population and GDP, temperature shows a more pronounced promoting effect on TN (r = 0.51), indicating a significant seasonal influence on nitrogen cycling.

Based on the above analysis, a multiple linear regression model for TN was constructed using SPSS, with ammonia nitrogen, temperature, barren land, and EC as independent variables. This model can be used to quantify management priorities and predict potential risks associated with nitrogen pollution. The resulting regression equation (R^2^ = 0.594) is:

TN = 0.761 × NH_3_-N + 0.355 × TEM − 0.248 × Barren land + 0.148 (2)

The standardized coefficient of ammonia nitrogen is the largest (0.761), making it the most important positively correlated driving factor for total nitrogen. This indicates that controlling ammonia nitrogen is the key to reducing total nitrogen. The coefficient of temperature is 0.355, indicating that rising temperatures promote nitrogen release, mineralization, and runoff migration. In the water source area, agricultural non-point source pollution should be controlled, and during high-temperature seasons, nitrogen pollution monitoring and reservoir discharge flow scheduling should be strengthened to reduce the risk of eutrophication outbreaks.

### 3.4. Simulation of Future Water Self-Purification Capacity in the Water Source Area

**Figure 7:**
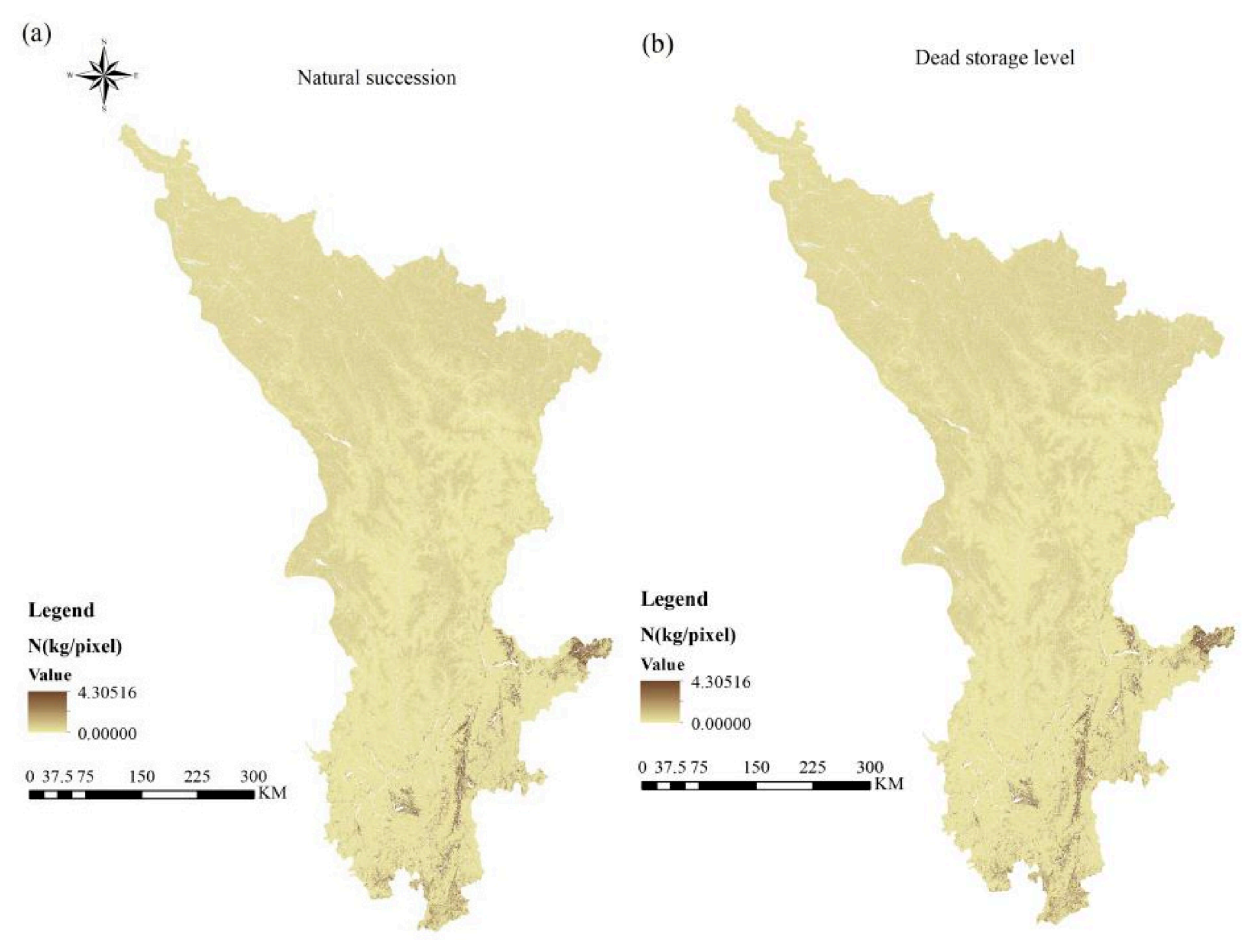

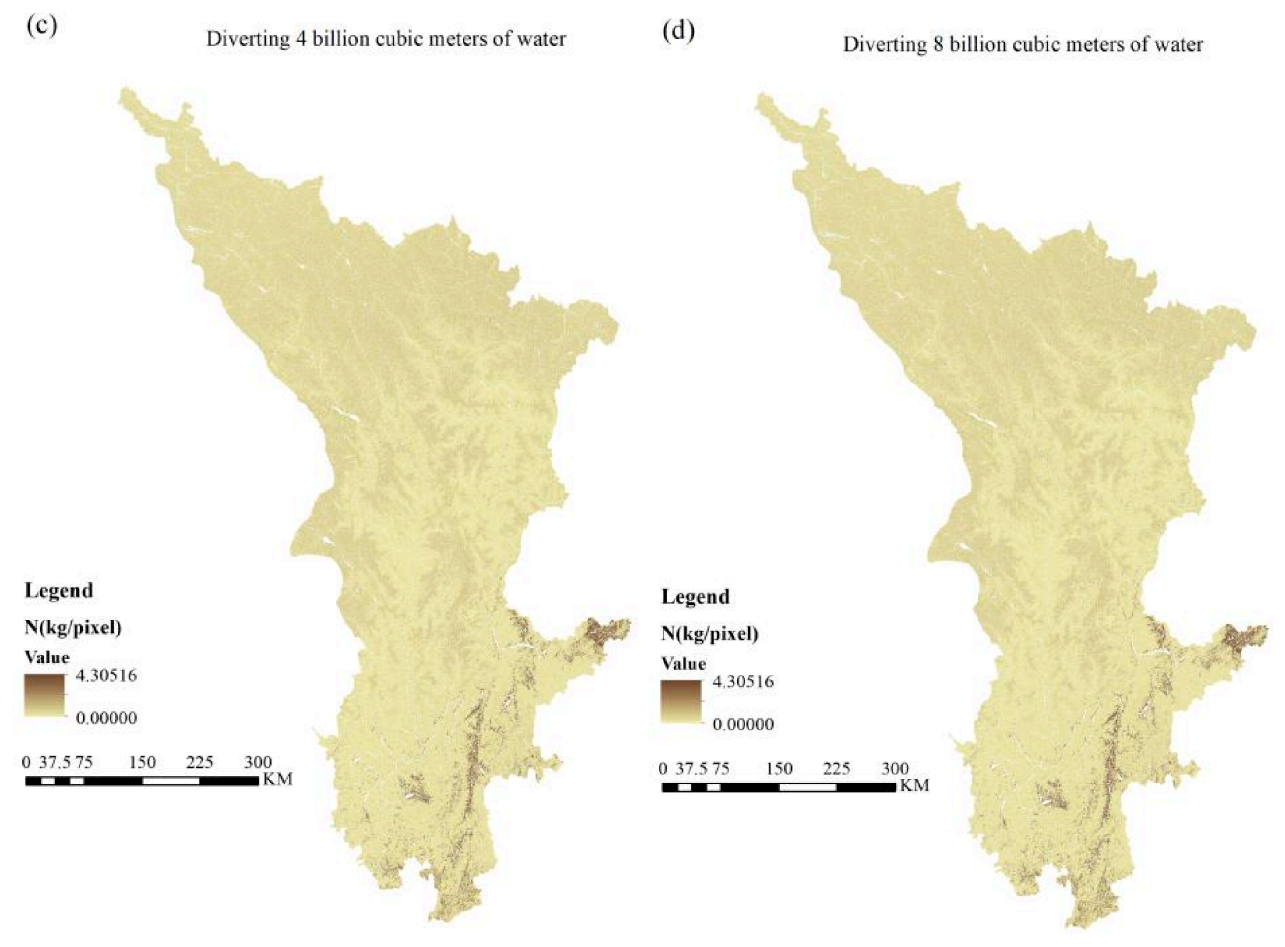
Nitrogen removal: (**a**) natural succession; (**b**) reservoir construction; (**c**) water diversion of 4 billion m^3^; (**d**) water diversion of 8 billion m^3^.

**Figure 8:**
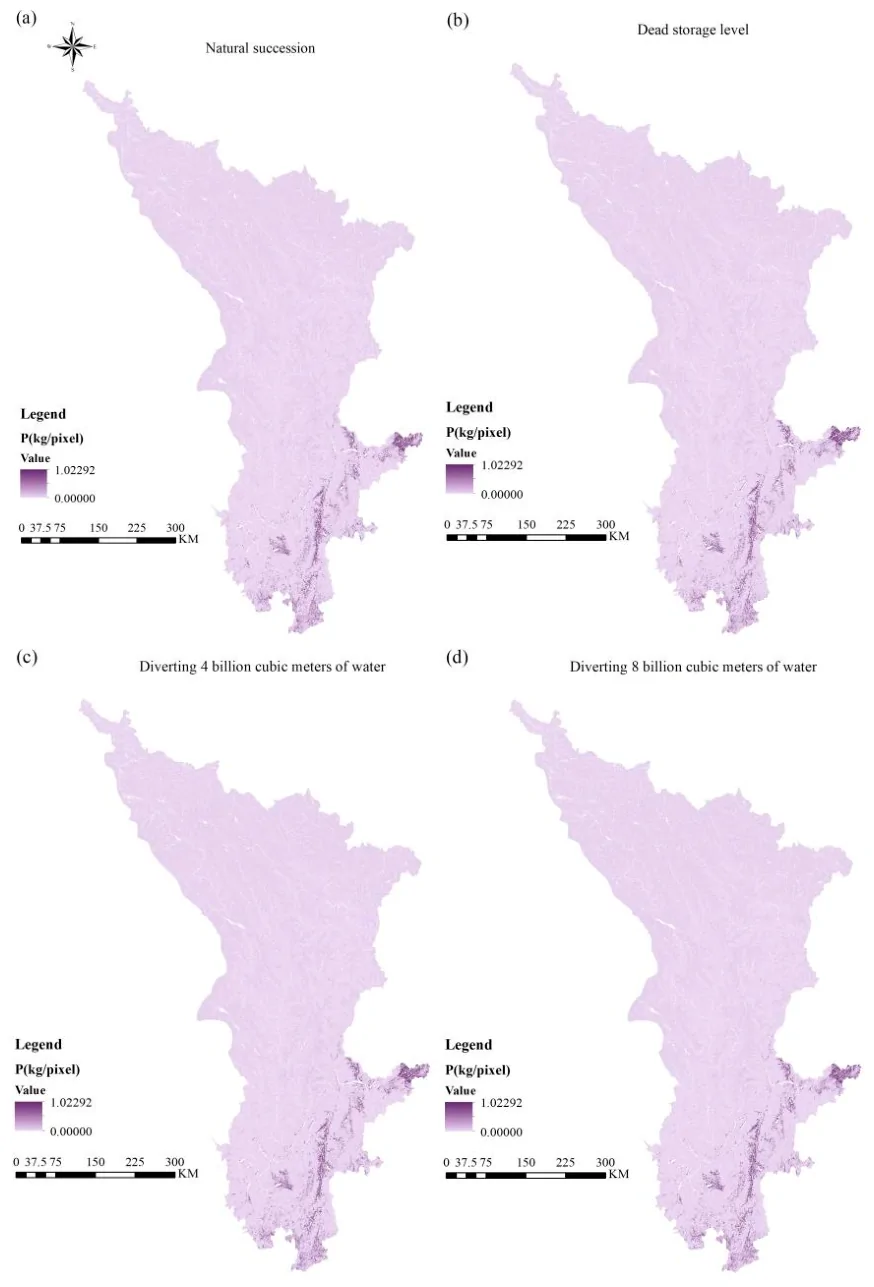
The phosphorus removal: (**a**) natural succession; (**b**) reservoir construction; (**c**) water diversion of 4 billion m^3^; (**d**) water diversion of 8 billion m^3^.

The PLUS model was employed to simulate the water self-purification service value in the water source area under four scenarios for 2050: natural succession, reservoir construction, water diversion of 4 billion m^3^, and water diversion of 8 billion m^3^. The nitrogen removal was shown in Figure 7a–d. The phosphorus removal was shown in Figure 8a–d. Under the natural succession scenario, nitrogen removal was 31,914 t and phosphorus was 2951 t; under the reservoir construction scenario, nitrogen removal was 31,909 t and the phosphorus was 2951 t; under the 4 billion m^3^ water diversion scenario, nitrogen removal was 31,970 t and the phosphorus was 2934 t; while with 8 billion m^3^ water diversion scenario, nitrogen removal was 31,766 t and the phosphorus was 2933 t. Compared with the natural scenario, reservoir construction reduced nitrogen by 4.9 t; the 4 billion m^3^ diversion reduced 144.1 t of nitrogen and phosphorus was 16.6 t; the 8 billion m^3^ diversion reduced 147.9 t of nitrogen and phosphorus was 18.4 t. After water diversion, the removal of nitrogen and phosphorus in the water source declined, and the removal of nitrogen was not linearly related to the amount of water diverted. The ecological service value of water self-purification under natural conditions was approximately 55.3 million yuan, and after reservoir construction it was 55.2 million yuan; compared with the natural condition, the 4 billion m^3^ diversion reduced it by 25.8 million yuan, and the 8 billion m^3^ diversion reduced it by 26.8 million yuan. This nonlinear response arises because the damaged area of grassland and other land use types does not expand proportionally with the volume of water diversion. The water quality in the source area is relatively good, and the impact of the project implementation on the water self-purification capacity is acceptable. It is necessary to further explore its effects on other factors.

## 4. Conclusions

This study evaluated the water self-purification capacity of the Western Route source area to support coordinated source protection and water-transfer benefits. Water quality in the upper reaches is generally good, though total nitrogen at some Dadu River sites exceeded 1.0 mg/L, with higher TN levels in the Dadu basin than in the Yalong basin, while total phosphorus remained low across both. The amount of nitrogen removal dominated purification services, being more than ten times that of phosphorus, which peaked in 2012 (34,654 t), while phosphorus peaked in 2017 (3306 t). Spatially, Garzê Prefecture contributed the most to water self-purification value, followed by Liangshan, Aba, and Golog. TN was primarily associated with barren land (r = 0.41) and grassland (r = 0.47), suggesting erosion and fertilized pastures as key sources. Under diversion scenarios, reservoir construction reduced the amount of nitrogen by 4.9 t; the 4 billion m^3^ and 8 billion m^3^ diversions reduced nitrogen by 144.1 t and 147.9 t, and phosphorus by 16.6 t and 18.4 t, respectively. Notably, the amount of nitrogen removal declined with diversion but showed no linear relationship with diversion volume, implying nonlinear ecological responses. These findings provide a quantitative basis for ecological compensation, diversion optimization, and green hydropower certification. This study offers baseline data, model frameworks, and scenario design templates, yet limitations in data and methods call for further progress in mechanism analysis, uncertainty evaluation, and multi-objective trade-offs. Future work can build upon this by incorporating refined monitoring, advanced algorithms, and diverse management needs to drive more scientific and accurate ecological impact assessments of water transfer projects.

## Data Availability

The original contributions presented in this study are included in the article/Supplementary Materials. Further inquiries can be directed to the corresponding author.

## Supplementary Materials

The following supporting information can be downloaded at https://www.mdpi.com/article/10.3390/toxics14100896/s1, Table S1: Water quality analysis of 18 water sampling points.
