# Supplementary material for: Spatiotemporal Patterns and Driving Factors of Water Self-Purification Capacity in the Source Area of the Western Route of the South-to-North Water Diversion
Source: Toxics. 2026 Oct 10;14(10):896. doi: 10.3390/toxics14100896 (PMC13709486; doi:10.3390/toxics14100896)
Supplement: Supplementary file 1 [file toxics-14-00896-s001.zip › toxics-4550173-supplementary.pdf]

Supplementary material

A total of 18 water sampling points was set up in the upper reaches of the Dadu and Yalong rivers within the source area, where water samples were collected for water quality analysis. The analytical results for pH, EC, TDS, COD, NH_3-N, TN, and TP were presented in Table S1, the values in the Table S1 are averaged.

Table S1. Water quality analysis of 18 water sampling points

Sampling point	Water quality indicator						
	pH	EC (us/cm)	TDS (mg/L)	COD (mg/L)	NH_3-N (mg/L)	TN (mg/L)	TP (mg/L)
1	8.56	391	195.5	1.03	0.22	0.32	0.010
2	7.92	274	137.1	1.64	0.22	0.64	0.002
3	8.14	377	189.1	0.55	0.17	0.66	0.005
4	8.23	307	153.5	0.75	0.27	1.02	0.007
5	8.52	315	157.8	2.06	0.22	1.17	0.016
6	8.47	299	149.9	0.92	0.12	1.03	0.011
7	7.92	146	73.0	7.01	0.07	0.49	0.085
8	8.05	211	105.3	1.02	0.07	0.82	0.137
9	7.84	239	121.0	5.29	0.01	0.14	0.03
10	7.87	246	114.0	4.57	0.11	0.29	0.018
11	7.93	264	134.5	5.07	0.14	0.64	0.018
12	7.76	159	80.4	5.30	0.11	0.45	0.018

13	7.89	238	124.0	4.47	0.10	0.47	0.018
14	8.34	405	205.0	3.41	0.12	0.54	0.014
15	8.4	412	206.0	3.97	0.11	0.45	0.025
16	8.16	273	136.5	4.06	0.11	0.54	0.014
17	8.02	274	136.8	3.31	0.10	0.34	0.018
18	8.08	244	122.2	4.65	0.11	0.51	0.021
